# Double posteromedial portal arthroscopy vs. other arthroscopic techniques for Baker's cyst: a systematic review and meta-analysis

**DOI:** 10.3389/fsurg.2026.1772431

**Published:** 2026-03-27

**Authors:** Fang Li, Xiaojun Fu, Juncheng Yu, Junhua Chen, Liming Xu, Jie Xiao

**Affiliations:** Department of Orthopedics, The First People’s Hospital of Jiande, Jiande, China

**Keywords:** arthroscopy, Baker's cyst, meta-analysis, popliteal cyst, posteromedial portal

## Abstract

**Background:**

Double posteromedial portal (DPP) arthroscopy has been proposed to improve cyst management in patients with Baker's cysts compared with other arthroscopic techniques. However, comparative evidence remains limited.

**Methods:**

A systematic review and meta-analysis was conducted according to PRISMA guidelines. Comparative clinical studies assessing DPP vs. non-DPP arthroscopic techniques for Baker's cysts were identified from PubMed, Embase, Scopus, Web of Science Core Collection and the Cochrane Library. The primary outcome was cyst recurrence or residual cyst at final follow-up. Secondary outcomes included Lysholm score, operative time, and overall complications. Pooled risk ratios (RRs) or mean differences (MDs) were calculated using fixed- or random-effects models depending on heterogeneity (I²). Sensitivity analyses were performed to assess result robustness.

**Results:**

Four comparative studies (all from China; total *n* = 232) were included. DPP arthroscopy showed a lower risk of cyst recurrence/residual cyst (RR = 0.17, 95% CI 0.04–0.74; RD = −0.08, 95% CI −0.14 to −0.02). Lysholm scores at final follow-up were similar between groups (MD = −0.65, 95% CI −2.14 to 0.84). Operative time was longer with DPP (MD = 13.20 min, 95% CI 6.16–20.24; I² = 91%), but remained longer after sensitivity analysis (MD = 10.02 min, 95% CI 7.09–12.96). Overall complications were higher with DPP (RR = 3.90, 95% CI 1.26–12.05; I² = 0%).

**Conclusion:**

DPP arthroscopy may reduce cysts recurrence compared with other arthroscopic approaches, but at the cost of longer operative time and higher complication rates. Evidence is limited to small, single-country studies with sparse recurrence events; further multicentre trials are needed.

## Introduction

Popliteal cysts, commonly known as Baker's cysts, represent a frequent clinical and imaging finding characterized by synovial fluid expansion of the gastrocnemius–semimembranosus bursa (1). They are seldom isolated phenomena and are typically associated with underlying intra-articular disorders such as meniscal tears, chondral degeneration, and osteoarthritis that drive synovitis and chronic effusion (2). Although ultrasonography is a reliable modality for confirming the diagnosis (3), its incremental value in routine management pathways can be variable, emphasizing that imaging findings must be integrated with clinical assessment (4). The strong association between symptomatic cysts and coexisting knee pathology underscores the principle that treatment should target the joint environment, not merely the cystic lesion itself (1, 5, 6).

The pathoanatomy of cyst persistence is attributed to a unidirectional valvular mechanism at the communication between the knee joint and the bursa, which permits fluid ingress but inhibits egress (1). Consequently, durable success requires interventions that mitigate the intra-articular fluid source and disrupt this valve mechanism (1, 5). Advances in arthroscopic technology have facilitated a posterior approach to the knee, with the adoption of posterior portals improving access to the posteromedial compartment for targeted management of the communication site (7).

In the surgical management of symptomatic or recurrent cysts, arthroscopic procedures have gained prominence over traditional open excision. Open surgery may be associated with recurrence, particularly when intra-articular drivers and the communication mechanism are not adequately addressed (6). In contrast, arthroscopy allows for concurrent treatment of intra-articular pathologies and definitive decompression of the cyst through a minimally invasive approach, a strategy supported by clinical series demonstrating low recurrence and good functional outcomes (5, 6). Within the spectrum of arthroscopic techniques, a persistent debate exists regarding the optimal handling of the cyst wall. Comparative studies have evaluated internal drainage with cyst wall preservation against resection, yet the balance between reducing recurrence and minimizing surgical morbidity remains unclear (8, 9).

As a technical evolution aimed at overcoming the challenges of posterior compartment arthroscopy, the double posteromedial portal (DPP) technique has been introduced. It is proposed to offer improved visualization and instrument triangulation, facilitating more complete management of the valvular communication and enabling cyst wall handling when indicated (10). Early comparative reports, primarily from Chinese institutions, have begun to assess DPP against conventional single-portal or other arthroscopic methods (11–14). However, the available comparative evidence is predominantly derived from Chinese single-centre cohorts, and the overall evidence base remains limited. Therefore, a systematic review and meta-analysis is needed to synthesise current data on DPP vs. other arthroscopic techniques and to clarify the consistency of reported efficacy and safety outcomes across studies.

## Methods

### Study design and reporting

Following the Preferred Reporting Items for Systematic Reviews and Meta-Analyses PRISMA 2020 guidelines (15), we performed a systematic review and meta-analysis using prespecified methods. The analysis protocol was prospectively registered in the PROSPERO International Register of Systematic Reviews (CRD420251268711). A protocol specifying eligibility criteria, outcomes, and analytical methods was developed *a priori*. Study screening, data extraction, and risk of bias assessment were performed independently by two reviewers; disagreements were resolved by discussion and, when necessary, adjudication by a third reviewer.

### Search strategy

A systematic search of PubMed, Embase, Scopus, Web of Science Core Collection, and the Cochrane Library was conducted from database inception to 30 November 2025. The strategy combined controlled vocabulary and free-text terms related to popliteal (Baker's) cyst, arthroscopy, and double posteromedial portal, together with terms describing comparator arthroscopic techniques. Searches were limited to English-language full-text articles. Full search strategies are provided in the Supplementary Material. Reference lists of eligible studies and relevant reviews were manually screened to identify additional studies.

### Eligibility criteria

Comparative clinical studies (randomized or non-randomized) were eligible if they enrolled patients with imaging-confirmed symptomatic Baker's (popliteal) cysts and compared double posteromedial portal (DPP) arthroscopy with other arthroscopic techniques not using DPP, and reported at least one prespecified outcome with extractable group-level data. Non-comparative case series, case reports, reviews, conference abstracts without full data, non-arthroscopic interventions, and purely open-surgery comparisons were excluded. When overlapping cohorts were suspected, the most informative report (largest sample size and/or most complete outcome reporting) was retained.

### Study selection

After de-duplication, two reviewers independently screened titles/abstracts followed by full-text review of potentially eligible reports. Reasons for full-text exclusion were recorded. Disagreements were resolved by consensus or third-party adjudication.

### Data extraction

Two reviewers extracted data independently using a piloted standardised form. Extracted items included: author, year, country, study design, sample size, intervention/comparator technique details, concomitant intra-articular procedures, follow-up duration, and outcome data (event counts; means and standard deviations). When multiple follow-up time points were reported, the longest available follow-up was extracted for meta-analysis.

### Outcomes

The primary outcome was the proportion of residual or recurrent popliteal Baker's cysts at final follow-up. Residual or recurrent cysts were defined primarily based on imaging assessment at final follow-up, using MRI or ultrasound as reported by each study, and were counted as events, whereas cyst disappearance or shrinkage was treated as non-events. Where reported, clinical grading systems such as the Rauschning–Lindgren system were recorded but were not used as the sole basis for event classification. When outcomes were reported at multiple follow-up time points, data from the longest available follow-up were used for meta-analysis.

Secondary outcomes comprised knee function at final follow-up assessed using the Lysholm score, with higher scores indicating better function, operative time for the index arthroscopic procedure, and overall postoperative complications defined as the occurrence of any complication as an event vs. no complication as a non-event. Where available, we sought to distinguish minor vs. major complications; however, severity grading and complication-type reporting were not standardised across studies, precluding reliable subgroup analyses. Studies that reported complications only at the overall cohort level without group-specific event counts were excluded from quantitative pooling for the complications outcome and were summarised narratively.

### Risk of bias assessment

Risk of bias for non-randomized comparative studies was assessed using the Newcastle–Ottawa Scale (NOS) across selection, comparability, and outcome domains (maximum 9 points) (16). Two reviewers assessed studies independently; disagreements were resolved by consensus or adjudication by a third reviewer.

### Statistical analysis

Meta-analyses were performed using Review Manager version 5.4. For continuous outcomes reported on the same scale, mean differences with 95% confidence intervals were calculated. Dichotomous outcomes were summarised as risk ratios with 95% confidence intervals. Fixed-effect models were used for the primary analyses when statistical heterogeneity was low and the number of studies was small. Statistical heterogeneity was assessed using Cochran's *Q* test and quantified with the I squared statistic, with *P* < 0.10 suggesting heterogeneity and I squared values greater than 50% indicating substantial heterogeneity.

When substantial heterogeneity was present, random-effects models were applied. Sensitivity analyses were conducted by sequentially excluding individual studies, and a leave-one-out approach was used for operative time to explore influential studies. For the primary outcome, robustness was additionally assessed using an alternative effect measure, odds ratio, and a random-effects model, with these analyses reported in the Supplementary Material. Absolute effects for dichotomous outcomes were additionally expressed as risk differences as a sensitivity analysis, and a continuity correction of 0.5 was applied for zero-event cells as implemented in the software. Publication bias was not assessed because fewer than 10 studies were available, making funnel plots and formal tests unreliable.

## Results

### Study selection and study characteristics

**Figure 1:**
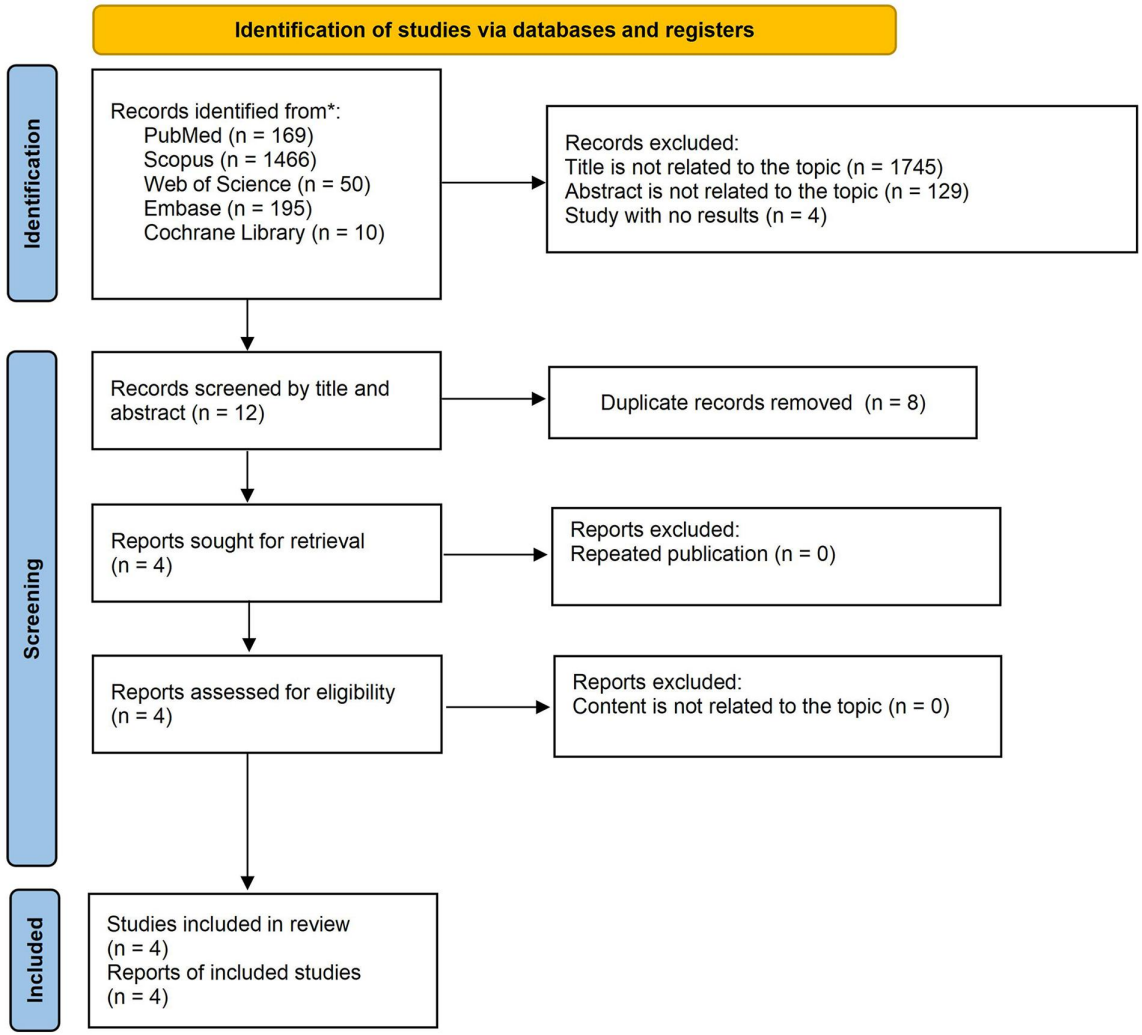
The PRISMA flow diagram displays the details of the selection process. *From: Page et al. (33).

Four comparative studies evaluating double posteromedial portals (DPP) vs. other arthroscopic techniques for symptomatic popliteal (Baker's) cysts were included in the quantitative synthesis (Figure 1) (11–14). Across these studies, a total of 227 participants contributed data to the primary outcome analysis (DPP, *n* = 118; non-DPP, *n* = 109). Key study and patient characteristics are summarised in Table 1, with detailed treatment characteristics and outcome data provided in Supplementary Table S1. Comparator techniques varied across studies, ranging from single posteromedial portal approaches to procedures differing in cyst wall management, which may partly explain heterogeneity in operative time and complication reporting. Risk-of-bias assessments for the non-randomised studies using the NOS indicated overall moderate-to-high methodological quality and are presented in Table 2. Key baseline variables relevant to confounding, including cyst size, severity of intra-articular pathology, and preoperative functional status, were not consistently reported across studies, limiting between-group comparability.

**Table 1:** Characteristics of included comparative arthroscopic studies for baker's cysts (DPP vs. other arthroscopic techniques).

Study (year)	Design	Intervention vs. Comparator	N (Int/Comp)	Age, years (mean ± SD)	Male, n/N (%)	Cyst outcome assessment	Follow-up (months)
Fu et al. (2025) (11)	Prospective RCT, single-centre	Internal drainage + cyst wall resection vs. internal drainage alone	30/30	55.50 ± 12.99 vs. 58.43 ± 14.14	8/30 (26.7%) vs. 6/30 (20.0%)	MRI at final follow-up (MRI-based residual/recurrence assessment)	∼12 (mean ± SD reported)
Guo et al. (2020) (12)	Retrospective comparative cohort	Two posteromedial portals vs. one posteromedial portal	28/25	52 ± 3 vs. 55 ± 3	15/28 (53.6%) vs. 12/25 (48.0%)	MRI-based assessment at follow-up	12–24 (reported range/approx.)
Ma et al. (2023) (13)	Retrospective case–control	Double posteromedial portal vs. single posteromedial portal	25/21	55.4 ± 8.6 vs. 54.4 ± 9.1	9/25 (36.0%) vs. 8/21 (38.1%)	MRI-based disappearance/shrinkage/recurrence at final follow-up	∼13 (mean ± SD by group)
Zhang et al. (2021) (14)	Retrospective case–control	Cyst wall resection vs. cyst wall preservation	38/35	51.8 ± 8.0 vs. 52.0 ± 7.9	14/38 (36.8%) vs. 13/35 (37.1%)	MRI outcome at last follow-up (persistence/recurrence vs. resolution)	∼24 (mean; range reported)

DPP, double posteromedial portal; MRI, magnetic resonance imaging; RCT, randomized controlled trial; SD, standard deviation; Int, intervention; Comp, comparator; n/N indicates number/total.

**Table 2:** The Newcastle–Ottawa scale (NOS) for assessing the quality of non-randomised studies included in this review.

Study	Year	Country	Type of Article	The Newcastle-Ottawa Scale (NOS)		
				Selection	Comparability	Exposure
Fu et.al (11)	2025	China	Prospective randomized controlled trial (single-centre)	***	***	***
Guo et.al (12)	2020	China	Retrospective comparative cohort study	***	**	***
Ma et.al (13)	2023	China	Retrospective comparative case–control study	***	**	***
Zhang et.al (14)	2021	China	Retrospective case–control comparative study	***	**	***

** = 2 points.

*** = 3 points.

### Primary outcome: residual or recurrent cyst at final follow-up

**Figure 2:**
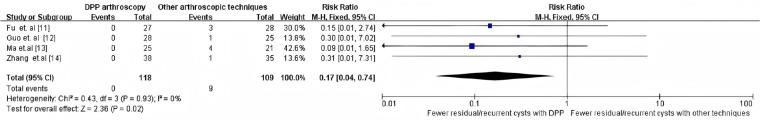
Residual or recurrent Baker's cyst at final follow-up comparing double posteromedial portal (DPP) arthroscopy versus other arthroscopic techniques: fixed-effect mantel–haenszel risk ratio (RR) meta-analysis shown as a forest plot. DPP, double posteromedial portal; RR, risk ratio; M-H, Mantel–Haenszel; CI, confidence interval.

Four comparative studies (DPP arthroscopy, *n* = 118; other arthroscopic techniques, *n* = 109) reported residual or recurrent cysts at the final follow-up (11–14). Across the included studies, no residual/recurrent cysts occurred in the DPP groups (0/118) compared with nine events in the comparator groups (9/109). Using a Mantel–Haenszel fixed-effect model, DPP was associated with a significantly lower risk of residual/recurrent cysts (RR 0.17, 95% CI 0.04–0.74; *P* = 0.02; I²=0%) (Figure 2). However, this estimate is based on sparse events with zero events in the DPP arms; thus the pooled RR depends on continuity correction and should be interpreted cautiously as potentially unstable.

**Figure 3:**
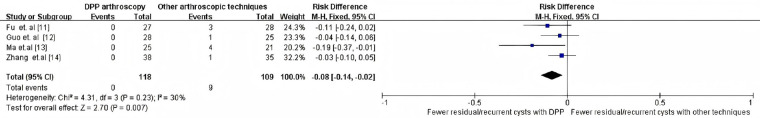
Residual or recurrent Baker's cyst at final follow-up comparing DPP arthroscopy versus other arthroscopic techniques: fixed-effect mantel–haenszel risk difference (RD) meta-analysis shown as a forest plot. DPP, double posteromedial portal; RD, risk difference; M-H, Mantel–Haenszel; CI, confidence interval.

When expressed as an absolute effect, DPP remained associated with fewer residual/recurrent cysts (RD −0.08, 95% CI −0.14 to −0.02; *P* = 0.007), corresponding to an absolute reduction of approximately 8% at the final follow-up, with low-to-moderate heterogeneity (I² = 30%) (Figure 3).

Findings were consistent in sensitivity analyses using an alternative effect measure (odds ratio, OR) and a random-effects model (Supplementary Figure S1–S2).

### Secondary outcomes

#### Knee function: lysholm score at final follow-up

**Figure 4:**
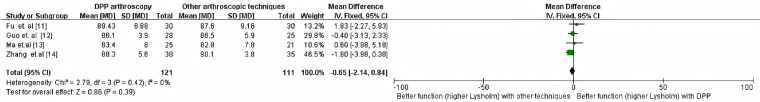
Lysholm knee function score at final follow-up comparing DPP arthroscopy versus other arthroscopic techniques: fixed-effect inverse-variance mean difference (MD) meta-analysis shown as a forest plot. DPP, double posteromedial portal; MD, mean difference; IV, inverse variance; CI, confidence interval.

Four comparative studies reported Lysholm scores at final follow-up (DPP arthroscopy, *n* = 121; other arthroscopic techniques, *n* = 111) (11–14). Fixed-effect meta-analysis showed no significant difference in Lysholm score between groups (MD −0.65, 95% CI −2.14 to 0.84; *P* = 0.39), with no evidence of heterogeneity (I² = 0%) (Figure 4).

#### Operative time

**Figure 5:**
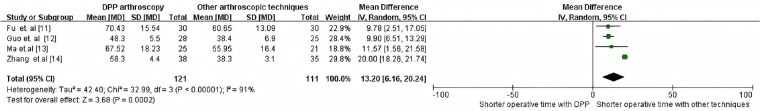
Operative time comparing DPP arthroscopy versus other arthroscopic techniques: random-effects inverse-variance mean difference (MD) meta-analysis shown as a forest plot. DPP, double posteromedial portal; MD, mean difference; IV, inverse variance; CI, confidence interval.

Four comparative studies reported operative time (DPP arthroscopy, *n* = 121; other arthroscopic techniques, *n* = 111) (11–14). Using a random-effects model, operative time was longer in the DPP group (MD 13.20 min, 95% CI 6.16–20.24; *P* = 0.0002), with substantial heterogeneity (I² = 91%) (Figure 5). In a leave-one-out sensitivity analysis, excluding the study by Zhang et al. (14) eliminated heterogeneity (I²=0%) and the direction of effect was unchanged (MD 10.02 min, 95% CI 7.09–12.96; *P* < 0.00001) (Supplementary Figure S3).

#### Overall complications

**Figure 6:**
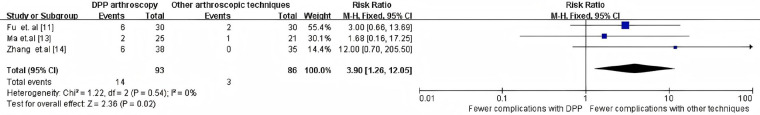
Overall postoperative complications comparing DPP arthroscopy versus other arthroscopic techniques: fixed-effect mantel–haenszel risk ratio (RR) meta-analysis shown as a forest plot. DPP, double posteromedial portal; RR, risk ratio; M-H, Mantel–Haenszel; CI, confidence interval.

Overall complications were reported in three studies (DPP arthroscopy, *n* = 93; other arthroscopic techniques, *n* = 86) (11, 13, 14). Fixed-effect meta-analysis showed a higher complication rate in the DPP group (RR 3.90, 95% CI 1.26–12.05; *P* = 0.02; I² = 0%) (Figure 6). Across studies, complication definitions were not standardised and events appeared predominantly minor and self-limited; therefore, conclusions regarding major complications cannot be reliably drawn. In an absolute-effect sensitivity analysis, DPP was also associated with a higher risk of complications (RD 0.12, 95% CI 0.03–0.20; *P* = 0.006; I² = 0%) (Supplementary Figure S4). One study was not included in the meta-analysis because complications were reported only at the overall cohort level without group-specific data (12).

## Discussion

DPP arthroscopy is a posterior-compartment–focused technique that improves visualization and instrument triangulation around the joint–bursa communication, potentially enabling more complete decompression and cyst control. However, the lack of a clear Lysholm advantage suggests that symptom relief and knee function are driven more by intra-articular pathology, concomitant procedures, and rehabilitation than by imaging-defined cyst persistence alone. Any anatomical benefit should therefore be balanced against longer operative time and postoperative event burden when selecting technique and counselling patients.

Prior evidence syntheses on Baker's cyst management have largely addressed broad strategy questions, including nonoperative vs. operative options and open excision vs. arthroscopic approaches (17, 18). Within arthroscopic management, the main comparative focus has been whether cyst wall preservation or resection yields better radiographic cyst outcomes and what perioperative costs may accompany more aggressive posterior work (8, 9, 19). Building on this background, our meta-analysis provides a more technique-specific synthesis by isolating double posteromedial portal (DPP) arthroscopy vs. other arthroscopic techniques and by jointly appraising cyst control at final follow-up alongside knee function and perioperative safety outcomes, enabling an explicitly decision-oriented interpretation of benefit vs. burden (11–14).

For reproducibility, DPP arthroscopy typically uses two posteromedial portals placed in the posteromedial compartment to establish a stable viewing portal and a working portal, enabling instrument triangulation toward the joint–bursa communication. Under arthroscopic visualization, the valvular slit is identified and enlarged to allow bidirectional drainage, and cyst wall management is performed selectively according to cyst morphology and surgeon preference. Key steps therefore include safe posteromedial portal placement, clear identification of the communication, controlled enlargement of the opening, and cautious handling of the cyst wall and adjacent neurovascular structures (10, 20, 21).

The lower risk of residual or recurrent cysts observed with the DPP technique is consistent with its proposed technical advantages and the established pathoanatomy of Baker's cysts. Persistence is generally attributed to a valve-like, unidirectional communication between the knee joint and the gastrocnemius–semimembranosus bursa, sustained by intra-articular pathology and effusion. Accordingly, contemporary syntheses emphasise that durable surgical success depends on addressing both the intra-articular source of fluid and the joint–bursa communication, rather than treating the cyst as an isolated lesion (1, 6, 17). Technical endoscopic reports further indicate that reliable identification and adequate enlargement of the valvular slit, together with selective cyst wall management when indicated, are key procedural steps intended to reduce residual or recurrent cysts (10, 22, 23). By improving visualization and enabling instrument triangulation in the posteromedial compartment, DPP may facilitate more consistent execution of these steps, providing a biologically plausible explanation for the lower residual or recurrent cyst risk seen in our pooled analysis (10).

In our pooled analysis, DPP arthroscopy was associated with a significantly lower risk of residual or recurrent cysts at final follow-up (RR 0.17, 95% CI 0.04–0.74; *P* = 0.02), based on an event distribution of 0/118 in the DPP arms vs. 9/109 in the comparator arms. This finding aligns directionally with the broader arthroscopic literature, which suggests that more comprehensive management of the joint–bursa communication and, when indicated, cyst wall management may improve postoperative cyst control. For instance, Su and colleagues reported MRI-defined persistence in 15% of patients treated with arthroscopic internal drainage alone, whereas no persistence was observed when cyst wall resection was added at follow-up (8). Similarly, a recent meta-analysis found that cyst wall resection was associated with better MRI-defined cyst outcomes, including fewer persistent or recurrent cysts, but at the cost of longer operative time and a higher complication signal—highlighting an efficacy–safety trade-off that parallels our secondary findings (9). Consistent with this pattern, Han et al. summarised earlier series and reported lower recurrence with cystectomy-based strategies alongside a higher complication burden compared with non-cystectomy approaches (19).

Taken together, these data support the clinical plausibility that DPP, by facilitating more thorough posteromedial work, could reduce residual or recurrent cysts, while underscoring the recurring theme of balancing efficacy against perioperative risk. Nevertheless, because the comparative evidence for DPP is still derived from a small number of single-centre cohorts in China, larger multicentre studies with standardised imaging endpoints are needed to further elucidate the magnitude and consistency of the observed association.

Although DPP was associated with a significantly lower rate of cyst recurrence, Lysholm scores at final follow-up were similar between groups (MD −0.65, 95% CI −2.14 to 0.84, I² = 0%). This dissociation between improved anatomical control and unchanged patient-reported function is clinically interpretable. First, the Lysholm score reflects global knee symptoms and function, which are predominantly determined by the intra-articular disease burden, the adequacy of concomitant arthroscopic management, and postoperative rehabilitation, rather than by a small residual cystic remnant on imaging. Consistent with this concept, residual cysts after arthroscopic treatment have been associated with degenerative cartilage lesions, underscoring the primacy of intra-articular pathology in shaping postoperative function (24). Second, residual or recurrent cysts are frequently defined radiographically and may be clinically silent. Baker's cysts are often asymptomatic when detected, and postoperative imaging may identify cyst persistence without a corresponding functional decrement, which helps explain why superior cyst control with DPP does not necessarily translate into a measurable Lysholm benefit (25, 26). This pattern aligns with prior comparisons in which more aggressive cyst management, such as cyst wall resection added to internal drainage, improved MRI outcomes without yielding superior functional scores (8, 9, 27), highlighting the distinction between anatomical endpoints and patient-important outcomes.

From a perioperative resource perspective, DPP arthroscopy required more operating time than comparator techniques, with a pooled mean difference of 13.20 min (95% CI 6.16–20.24; *P* = 0.0002) under a random-effects model (11–14). A leave-one-out analysis suggested that the Zhang cohort was the major driver of this variability, since excluding it did not materially change the overall interpretation and the time difference remained clinically evident, with a mean difference of 10.02 min (95% CI 7.09–12.96; *P* < 0.00001) (13). This finding is biologically and procedurally plausible because DPP adds posterior compartment work that is not always required in single-portal approaches, including creation of an additional posteromedial portal, establishment of a stable working view, and instrument triangulation to enlarge the joint–bursa communication and perform more complete cyst wall handling when indicated (10, 20, 21). In the included comparative DPP cohort, the authors explicitly noted that operative duration depended on surgeon experience and the technical difficulty of cyst wall resection, and that DPP typically took longer because an additional auxiliary posteromedial portal had to be established (12). Beyond portal number alone, differences in how aggressively surgeons pursue cyst wall debridement or cystectomy, as well as variations in cyst morphology and concomitant intra-articular procedures, are all credible clinical explanations for between-study variability in operative time, and they may also explain why the Zhang study behaved as an outlier without implying methodological flaws (9, 19, 28, 29). However, because these contributors were not uniformly reported at the study level, we could not formally test their effects, and the explanation for heterogeneity remains partly speculative. Consistent with this, prior technical and comparative reports describe longer operative workflows when posterior portals are added and when procedures escalate from drainage toward more complete cyst wall work or combined posterior approaches (10, 20, 21, 28, 29).

Safety is an important counterweight to the improved cyst control observed with DPP. Across the three studies that reported group-stratified data, overall postoperative complications were more frequent after DPP (RR 3.90, 95% CI 1.26–12.05; *P* = 0.02) (11, 13, 14). This signal is clinically plausible and appears most consistent with an increase in mild, self-limited events. The additional portal and more extensive posterior-compartment work required by DPP, which together with the longer operative time may predispose to fluid extravasation, localized swelling, ecchymosis, or small hematomas, were noted in the included cohorts (11, 13, 14). Importantly, these studies did not report a clear increase in major complications such as neurovascular injury, deep infection, or venous thromboembolism with DPP. Nevertheless, serious neurovascular events after knee arthroscopy, including popliteal artery injury, are rare but well documented and can be consequential, underscoring the need for vigilance when operating in the posterior compartment (18). Neurovascular complications have also been reported in surgical cohorts for popliteal cysts, although they appear uncommon (30). The interpretation of this finding requires caution because complication definitions and reporting thresholds likely varied across studies, including whether minor bruising or oozing was systematically recorded and the follow-up window used for ascertainment. Furthermore, the evidence base is incomplete because one otherwise eligible comparative study did not provide group-specific complication counts and therefore could not be included in the pooled analysis, creating potential for information bias (12). In summary, current data suggest an efficacy–safety trade-off in which DPP may increase the risk of predominantly minor complications, but they are insufficient to determine whether the risk of serious complications is truly higher; future studies should adopt standardised complication definitions and severity grading, ideally using a Clavien–Dindo–based framework, to enable consistent minor-vs.-major reporting and robust safety comparisons (31, 32).

From a clinical perspective, DPP may be most relevant when maximizing postoperative cyst control is a priority, particularly in symptomatic patients in whom persistent or recurrent cysts drive posterior knee fullness, repeat imaging, or follow-up burden. It may be particularly considered for symptomatic recurrent or persistent cysts, large or multiloculated cysts, or cases where single-portal access to the communication may be limited, although these subgroup considerations require prospective validation. However, because knee function at final follow-up was similar between techniques, the incremental value of DPP may be primarily anatomical rather than functional, and any potential advantage in cyst control should be weighed against longer operative workflows and a higher overall complication signal. This trade-off resembles patterns observed in broader arthroscopic comparisons, where more extensive cyst-directed procedures improved imaging-defined cyst outcomes but were associated with longer operative time and more complications (8, 9, 19). Therefore, technique selection is best individualized, integrating patient preferences regarding recurrence surveillance and tolerance of minor perioperative events with surgeon experience in posteromedial portals and posterior compartment work; given the small, single-centre comparative evidence base, these implications should be interpreted cautiously rather than as a recommendation for routine DPP use (10–14).

Our study has several strengths. First, we applied prespecified outcome definitions and consistently extracted data at final follow-up, enhancing cross-study comparability and clinical relevance. Second, presenting both relative and absolute effects for the primary outcome by reporting risk ratios alongside risk differences facilitates clinical interpretation. Third, sensitivity analyses using an alternative effect measure and a random-effects model yielded consistent results, supporting the robustness of the primary finding. Finally, a leave-one-out analysis identified the main driver of heterogeneity for operative time, strengthening the interpretability of this perioperative outcome.

This study has several inherent limitations. First, the evidence base is small, with only four studies and few events; zero-event cells required continuity correction, making the recurrence estimate statistically fragile due to sparse events and zero events in the DPP groups. All data were derived from single-centre Chinese cohorts, which limits statistical robustness and generalisability. This may reflect the current geographic concentration of the published comparative evidence for DPP. In addition, limiting inclusion to English-language full texts may have excluded relevant non-English studies, further limiting geographic representativeness. Second, most included studies were non-randomised, carrying risks of selection bias and residual confounding. Residual confounding may reflect differences in cyst morphology, intra-articular pathology and concomitant procedures, and surgeon experience. Third, heterogeneity across comparator techniques and outcome definitions, including imaging criteria for residual or recurrent cysts and complication ascertainment, complicates direct comparisons and may introduce measurement bias; future studies should apply prespecified, standardised radiological criteria. Finally, the limited number of studies precluded a formal assessment of publication bias.

In conclusion, this meta-analysis suggests that DPP arthroscopy is associated with fewer residual or recurrent cysts at final follow-up and similar postoperative knee function, but at the cost of longer operative time and a higher overall complication risk compared with other arthroscopic techniques. As the current evidence is based on small, non-randomized, single-centre Chinese cohorts, these findings should be interpreted cautiously. Larger multicentre studies with standardised outcome definitions and longer follow-up are warranted to better define the net clinical benefit and generalisability of the DPP technique.

## Supplementary material

SUPPLEMENTARY FIGURE S1 Residual or recurrent Baker's cyst at final follow-up comparing DPP arthroscopy versus other arthroscopic techniques: fixed-effect Mantel-Haenszel odds ratio (OR) sensitivity analysis shown as a forest plot. DPP, double posteromedial portal; OR, odds ratio; M-H, Mantel-Haenszel; CI, confidence interval.

SUPPLEMENTARY FIGURE S2 Residual or recurrent Baker's cyst at final follow-up comparing DPP arthroscopy versus other arthroscopic techniques: random-effects Mantel–Haenszel risk ratio (RR) sensitivity analysis shown as a forest plot. DPP, double posteromedial portal; RR, risk ratio; M-H, Mantel–Haenszel; CI, confidence interval.

SUPPLEMENTARY FIGURE S3 Operative time comparing DPP arthroscopy versus other arthroscopic techniques: fixed-effect inverse-variance mean difference (MD) leave-one-out sensitivity analysis excluding the Zhang cohort, shown as a forest plot. DPP, double posteromedial portal; MD, mean difference; IV, inverse variance; CI, confidence interval.

SUPPLEMENTARY FIGURE S4 Overall postoperative complications comparing DPP arthroscopy versus other arthroscopic techniques: fixed-effect Mantel–Haenszel risk difference (RD) sensitivity analysis shown as a forest plot. DPP, double posteromedial portal; RD, risk difference; M-H, Mantel–Haenszel; CI, confidence interval.
