# Supplementary figures and images for: Double posteromedial portal arthroscopy vs. other arthroscopic techniques for Baker's cyst: a systematic review and meta-analysis
Source: Front Surg. 2026 Mar 27;13:1772431. doi: 10.3389/fsurg.2026.1772431 (PMC13066308; doi:10.3389/fsurg.2026.1772431)

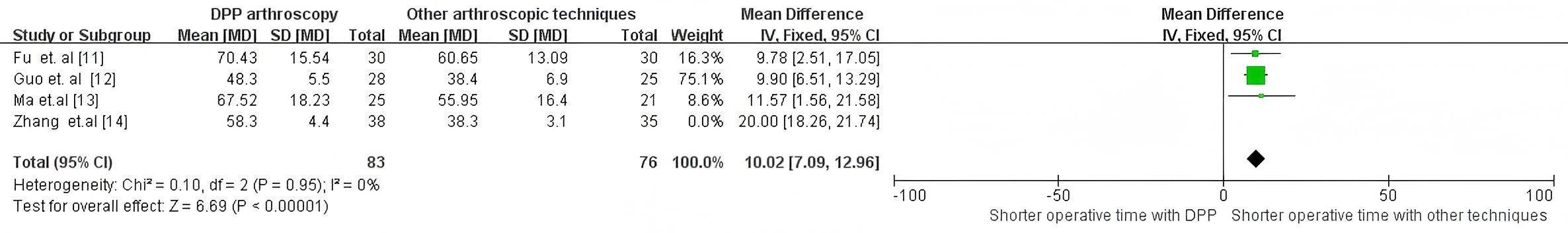

Supplement: SUPPLEMENTARY FIGURE S1 — Residual or recurrent Baker's cyst at final follow-up comparing DPP arthroscopy versus other arthroscopic techniques: fixed-effect Mantel-Haenszel odds ratio (OR) sensitivity analysis shown as a forest plot. DPP, double posteromedial portal; OR, odds ratio; M-H, Mantel-Haenszel; CI, confidence interval. [file Image1.jpeg]

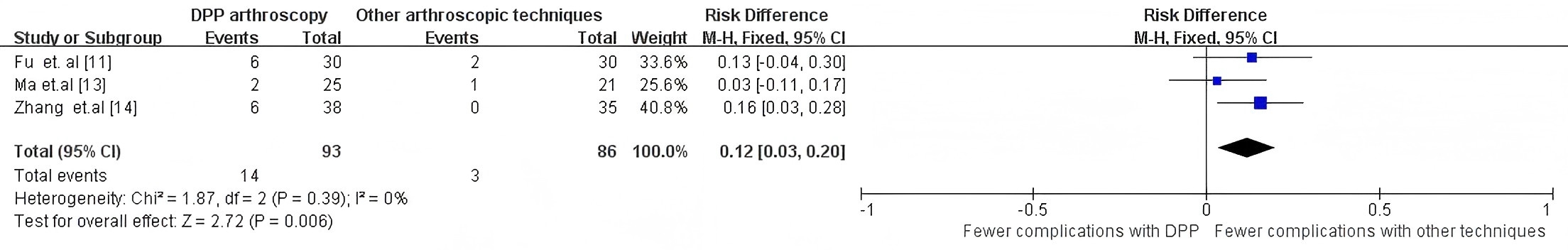

Supplement: SUPPLEMENTARY FIGURE S2 — Residual or recurrent Baker's cyst at final follow-up comparing DPP arthroscopy versus other arthroscopic techniques: random-effects Mantel–Haenszel risk ratio (RR) sensitivity analysis shown as a forest plot. DPP, double posteromedial portal; RR, risk ratio; M-H, Mantel–Haenszel; CI, confidence interval. [file Image2.jpeg]

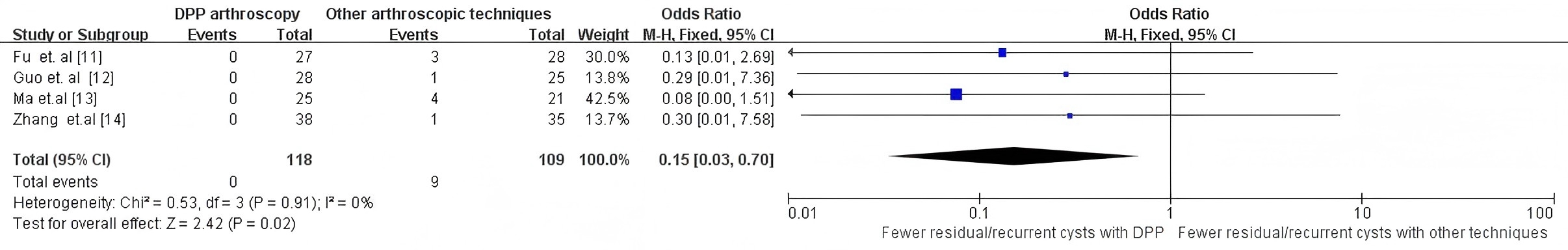

Supplement: SUPPLEMENTARY FIGURE S3 — Operative time comparing DPP arthroscopy versus other arthroscopic techniques: fixed-effect inverse-variance mean difference (MD) leave-one-out sensitivity analysis excluding the Zhang cohort, shown as a forest plot. DPP, double posteromedial portal; MD, mean difference; IV, inverse variance; CI, confidence interval. [file Image3.jpeg]

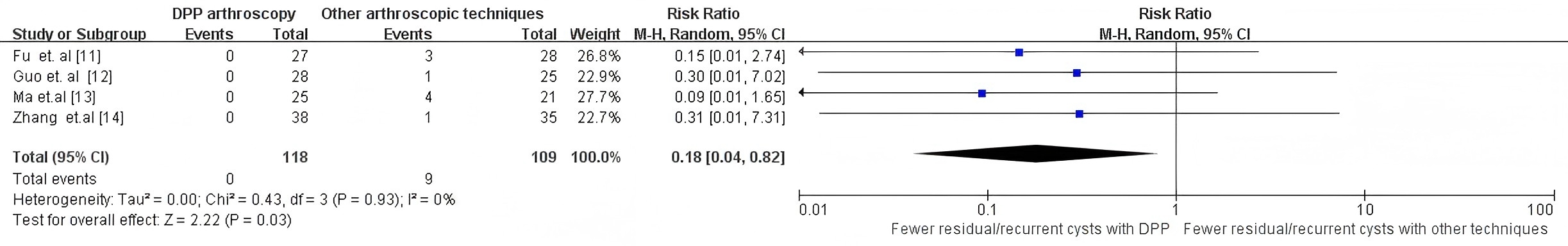

Supplement: SUPPLEMENTARY FIGURE S4 — Overall postoperative complications comparing DPP arthroscopy versus other arthroscopic techniques: fixed-effect Mantel–Haenszel risk difference (RD) sensitivity analysis shown as a forest plot. DPP, double posteromedial portal; RD, risk difference; M-H, Mantel–Haenszel; CI, confidence interval. [file Image4.jpeg]
