# Supplementary material for: Double posteromedial portal arthroscopy vs. other arthroscopic techniques for Baker's cyst: a systematic review and meta-analysis
Source: Front Surg. 2026 Mar 27;13:1772431. doi: 10.3389/fsurg.2026.1772431 (PMC13066308; doi:10.3389/fsurg.2026.1772431)
Supplement: Supplementary file 5 [file Table1.docx]

| Supplementary Table S1. Surgical techniques and key outcomes of included comparative studies of double posteromedial portal (DPP) arthroscopy versus other arthroscopic techniques for Baker’s cysts | | | | | | | | | | |
| --- | --- | --- | --- | --- | --- | --- | --- | --- | --- | --- |
| Study (year) | Design | DPP arthroscopic technique | Comparator arthroscopic technique | Concomitant intra-articular procedures | Definition of recurrence / residual cyst | Recurrence / residual cyst at final follow-up – DPP | Recurrence / residual cyst at final follow-up – Comparator | Functional outcomes (scale & main result) | Pain outcomes (scale & main result) | Complications and re-interventions |
| Fu et al (2025) [11] | Prospective RCT, single-centre | Arthroscopic internal drainage (AID) + complete cyst wall resection via double posteromedial portals (standard posteromedial [PM] + high PM); 30° arthroscope; shaver in high PM | AID via a single posteromedial portal without cyst wall resection | Treated intra-articular lesions as indicated (meniscal tears, cartilage lesions, loose bodies, synovial plica) | MRI-based residual ratio = postoperative/preoperative cyst volume; 0–<5% disappeared, 5–<30% shrunk, 30–<80% persisted, ≥80% recurred; “persisted” or “recurred” counted as residual/recurrence | 0/27 (0%) persistent or recurrent cyst | 3/28 (10.7%) persistent cysts | Lysholm and Rauschning–Lindgren scores improved in both groups; no significant between-group difference | VAS on postoperative days 1–3: pain decreased similarly in both groups; no significant between-group difference | No neurovascular injury, DVT or infection; transient subcutaneous ecchymosis: 6/30 (DPP) vs 2/30 (AID); no re-operations |
| Guo et al (2020) [12] | Retrospective comparative cohort | Two posteromedial portals (TPP): 30° arthroscope; capsular fold resected; valvular slit enlarged; cyst wall resected with low-suction shaver | One posteromedial portal (OPP): capsular fold resected; valvular slit enlarged using shaver (cyst wall not resected) | Routine arthroscopy to treat intra-articular pathology as indicated (e.g., cartilage lesions, meniscus tears, synovitis, loose bodies) | Follow-up MRI ≥12 months; recurrence defined as Rauschning–Lindgren grade ≥ II | 0/28 (0%) recurrent (grade ≥ II); MRI: disappeared 23, reduced 5 | 1/25 (4%) recurrent (grade ≥ II); MRI: disappeared 17, reduced 8 | Lysholm score at final follow-up: no significant between-group difference | Not reported | Complications reported overall only: 2/53 (3.8%) (1 deep vein thrombosis; 1 hypoesthesia); both recovered; no re-intervention reported |
| Ma et al (2023) [13] | Retrospective case–control | Double posteromedial portal (DPP): two PM portals with routine anterolateral/anteromedial portals; 30° lens; valvular slit enlarged; cyst wall resected with shaver via auxiliary PM portal | Single posteromedial portal (SPP): one PM portal with routine anterolateral/anteromedial portals; valvular slit enlarged; no auxiliary PM portal and no cyst wall resection | Routine arthroscopy to treat intra-articular pathology as indicated (degenerative cartilage damage, medial/lateral meniscal tears, synovitis, loose bodies) | Final follow-up (~1 year); MRI used to assess cyst outcome (disappearance/shrinkage/recurrence); recurrence defined by MRI | 0/25 (0%) recurrence; MRI: disappearance 17, shrinkage 8 | 4/21 (19.0%) recurrence; MRI: disappearance 9, shrinkage 8, recurrence 4 | Lysholm score: improved in both groups; no difference at final follow-up (83.4±8.0 vs 82.8±7.8; P=0.789) | Not reported | Fluid infiltration: 2/25 (8.0%) vs 1/21 (4.8%); no re-interventions reported |
| Zhang et al (2021) [14] | Retrospective case–control | Arthroscopic internal drainage (AID) + cyst wall resection (CWR) via standard PM portal plus additional high PM portal (HPM); 30° arthroscope; cyst wall resected with shaver after internal drainage | AID + cyst wall preservation (CWP) via single PM portal; cyst wall preserved | Routine anterolateral/anteromedial arthroscopy to treat intra-articular lesions as indicated (e.g., meniscus tear, cartilage lesion, synovitis, loose bodies) | Last follow-up MRI: cyst outcome recorded as disappeared/shrunk/persisted; “persisted” defined as residual/recurrence | 0/38 (0%) persisted (recurrence); MRI: disappeared 25, shrunk 13 | 1/35 (2.9%) persisted (recurrence); MRI: disappeared 22, shrunk 12, persisted 1 | Rauschning–Lindgren grade and Lysholm score: no between-group difference at final follow-up; Lysholm 88.3±5.6 (CWR) vs 90.1±3.8 (CWP), P=0.071; R–L grade P=0.630 | Not reported separately (VAS assessed within R–L grade) | Complications: 6/38 (15.8%) (fluid infiltration under gastrocnemius 3/38 [7.9%], hematoma 3/38 [7.9%]) vs 0/35 (0%); no serious neurovascular complications; no re-interventions reported |
| AID, arthroscopic internal drainage; CWP, cyst wall preservation; CWR, cyst wall resection; DPP, double posteromedial portal; DVT, deep vein thrombosis; HPM, high posteromedial; MRI, magnetic resonance imaging; OPP, one posteromedial portal; PM, posteromedial; R–L, Rauschning–Lindgren; SPP, single posteromedial portal; TPP, two posteromedial portals; VAS, visual analogue scale. | | | | | | | | | | |
