# Supplementary material for: Double posteromedial portal arthroscopy vs. other arthroscopic techniques for Baker's cyst: a systematic review and meta-analysis
Source: Front Surg. 2026 Mar 27;13:1772431. doi: 10.3389/fsurg.2026.1772431 (PMC13066308; doi:10.3389/fsurg.2026.1772431)
Supplement: Supplementary file 6 [file Supplementaryfile1.docx]

## Supplementary Methods: Literature search strategy

We systematically searched PubMed, Web of Science Core Collection, Scopus, Embase and the Cochrane Library for studies evaluating arthroscopic treatment of popliteal (Baker’s) cysts. Searches covered all years from database inception to **30 November 2025**. Only articles published in English were considered.

The search strategies combined terms related to popliteal (Baker’s) cysts and arthroscopic procedures. The full search strings for each database are reported below for transparency and reproducibility.

### 1. PubMed

**Database:** PubMed (MEDLINE)
**Date range:** From inception to 30 November 2025
**Search fields:** MeSH terms and Title/Abstract

**Concept #1 – Popliteal (Baker’s) cyst**
"Popliteal Cyst"[Mesh] OR "popliteal cyst"[tiab] OR "popliteal cysts"[tiab] OR "Baker cyst"[tiab] OR "Baker's cyst"[tiab] OR "Bakers cyst"[tiab] OR "Baker cysts"[tiab] OR "gastrocnemius-semimembranosus bursa"[tiab] OR "gastrocnemius semimembranosus bursa"[tiab]

**Concept #2 – Arthroscopy**
"Arthroscopy"[Mesh] OR arthroscop*[tiab] OR "arthroscopic surgery"[tiab] OR "arthroscopic treatment"[tiab] OR "arthroscopic management"[tiab] OR "arthroscopic procedure"[tiab] OR "arthroscopic procedures"[tiab]

**Final PubMed search**
#1 AND #2

### 2. Web of Science Core Collection

**Database:** Web of Science Core Collection
**Search field:** Topic (TS)
**Search date:** 30 November 2025

**Step 1 – Popliteal (Baker’s) cyst concept (#1)**
TS=("popliteal cyst" OR "popliteal cysts" OR "Baker cyst" OR "Baker's cyst" OR "Baker cysts" OR "Baker's popliteal cyst" OR "popliteal synovial cyst")

**Step 2 – Arthroscopy concept (#2)**
TS=(arthroscop* OR "arthroscopic surgery" OR "arthroscopic treatment" OR "arthroscopic management" OR "arthroscopic procedure" OR "arthroscopic procedures")

**Final Web of Science search**
#1 AND #2

### 3. Scopus

**Database:** Scopus
**Search date:** 30 November 2025

**Search string**
TITLE-ABS-KEY("popliteal cyst" OR "popliteal cysts" OR "Baker cyst" OR "Baker's cyst" OR "Baker cysts" OR "Baker's popliteal cyst" OR "popliteal synovial cyst*")
AND
TITLE-ABS-KEY(arthroscop* OR "arthroscopic surgery" OR "arthroscopic treatment" OR "arthroscopic management" OR "arthroscopic procedure" OR "arthroscopic procedures")

### 4. Embase

**Database:** Embase
**Search date:** 30 November 2025

**Step 1 – Popliteal (Baker’s) cyst concept (#1)**
('popliteal cyst' OR 'popliteal cysts' OR 'baker* cyst*' OR 'popliteal synovial cyst*')

**Step 2 – Arthroscopy concept (#2)**
(arthroscop* OR 'arthroscopic surgery' OR 'arthroscopic treatment' OR 'arthroscopic management' OR 'arthroscopic procedure' OR 'arthroscopic procedures')

**Final Embase search**
#1 AND #2

### 5. Cochrane Library

**Database:** Cochrane Library (CENTRAL and other Cochrane databases)
**Search date:** 30 November 2025

**Step 1 – Popliteal (Baker’s) cyst concept (#1)**
("popliteal cyst" OR "popliteal cysts" OR "Baker cyst" OR "Baker's cyst" OR "Baker cysts" OR "Baker's popliteal cyst" OR "popliteal synovial cyst")

**Step 2 – Arthroscopy concept (#2)**
(arthroscop* OR "arthroscopic surgery" OR "arthroscopic treatment" OR "arthroscopic management" OR "arthroscopic procedure" OR "arthroscopic procedures")

**Final Cochrane Library search**
#1 AND #2
